# Remotely connected: patient and clinician video care experiences in secondary mental health services during COVID-19, including future preferences

**DOI:** 10.1192/bjo.2021.178

**Published:** 2021-06-18

**Authors:** Lamiya Samad, Bonnie Teague, Karen Moreira, Sophie Bagge, Khalifa Elzubeir, Jonathan Wilson

**Affiliations:** 1 Norfolk and Suffolk NHS Foundation Trust, Medical Adviser - represent UCL Great Ormond Street Institute of Child Health, to British Paediatric Surveillance Unit, Royal College of Paediatrics and Child Health; 2 Norfolk and Suffolk NHS Foundation Trust, University of East Anglia; 3 Norfolk and Suffolk NHS Foundation Trust; Emma Marriott, Norfolk and Suffolk NHS Foundation Trust; 4 Norfolk and Suffolk NHS Foundation Trust; 5 Norfolk and Suffolk NHS Foundation Trust; 6 Norfolk and Suffolk NHS Foundation Trust, University of East Anglia

## Abstract

**Aims:**

Video-delivered care is a rapidly emerging area with potential to transform assessment and treatment strategies. The coronavirus (COVID-19) pandemic has accelerated these changes. Limited evidence exists for experiences of video care in secondary mental health services. We aimed to assess the acceptability of video care in mental health clinical practice during COVID-19.

**Method:**

Structured questionnaires were developed with the help of patients and clinicians. The patient experience questionnaire was built into video sessions and completed online, using the Attend Anywhere (AA) platform from July 2020 to March 2021. A Trust-wide clinician views and experiences survey was conducted from July 2020 to October 2020. Descriptive analysis was performed using SPSS (version 27.0).

**Result:**

Of 1,296 patients who completed the online feedback, the majority provided positive feedback for all aspects of video care. Most patients felt their needs were met (90%) and were supported (93%) during the video call. Positive experiences were informed by clinicians’ communication skills. For future appointments, just over half (51.7%) of patients preferred using video calls, followed by face-to-face (33%). Future video preference was informed by reasons reducing social anxiety and practical aspects such as child/carer needs, physical disability and travel.

Of 252 clinicians completing the survey, 161 (64.7%) had used video for remote care delivery. Clinicians also provided positive feedback, with Microsoft-teams as the preferred platform. Most clinicians felt the therapeutic relationship (76.4%) and privacy (78.7%) were maintained using video. While 73% felt there were no safeguarding issues that impacted adversely, 30% felt that care quality was affected, and (69.9%) reported limited visual cues for video calls. Most clinicians (73%) felt confident about clinical decision-making remotely, though there were areas where clinicians felt less confident, such as assessing patients’ appearance and behaviour. Additionally, compared with face-to-face, video consultations seemed to be effective for social anxiety, but less so for Autism spectrum disorders, and with no perceived difference for depression or self harm. For future, more clinicians preferred face-to-face (40.1%) than video care (36.1%).

**Conclusion:**

Mental health care delivered remotely via video is experienced positively by patients and clinicians alike. However, clinicians felt that quality of care is impacted, and additional remote clinical skills training may be beneficial. Going forward, there is acceptability for the use of video care in routine mental health practice for certain mental health presentations.

